# Development and validation of a pre-hospital “Red Flag” alert for activation of intra-hospital haemorrhage control response in blunt trauma

**DOI:** 10.1186/s13054-018-2026-9

**Published:** 2018-05-05

**Authors:** Sophie Rym Hamada, Anne Rosa, Tobias Gauss, Jean-Philippe Desclefs, Mathieu Raux, Anatole Harrois, Arnaud Follin, Fabrice Cook, Mathieu Boutonnet, the Traumabase® Group, Arie Attias, Sylvain Ausset, Gilles Dhonneur, Olivier Langeron, Catherine Paugam-Burtz, Romain Pirracchio, Bruno Riou, Guillaume de St Maurice, Bernard Vigué, Alexandra Rouquette, Jacques Duranteau

**Affiliations:** 1 Université Paris Sud, Department of Anesthesiology and Critical Care, AP-HP, Bicêtre Hôpitaux Universitaires Paris Sud, 78 rue du Général Leclerc, F-94275 Le Kremlin Bicêtre, France; 2 CESP, INSERM, Université Paris-Sud, UVSQ, Université Paris-Saclay, Paris, CESP, INSERM, Maison de Solenn, 97 boulevard de Port-Royal, 75014 Paris, France; 3 Université Paris Sud, Department of Anesthesiology and Critical Care, Assistance Publique-Hôpitaux de Paris (AP-HP), Béclère Hôpitaux Universitaires Paris Sud, 157 rue de la porte de Trivaux, 92140 Clamart, France; 4 Hôpitaux Universitaires Paris Nord Val de Seine, Department of Anesthesiology and Critical Care, AP-HP, Beaujon, 100 avenue du Général Leclerc, 92110 Clichy, France; 5 Centre Hospitalier Sud-Francilien, SAMU 91 Emergency Department, 40 avenue Serge Dassault, 91100 Corbeil-Essonnes, France; 6 Sorbonne Université and Department of Anesthesiology and Critical Care, AP-HP, Hôpitaux Universitaires Pitié-Salpêtrière, 47–83 Boulevard de l’Hôpital, 75013 Paris, France; 7 Université Paris Descartes, Department of Anesthesiology and Critical Care, AP-HP, Hôpital Européen Georges Pompidou—Hôpitaux Universitaires Paris Ouest, 20 rue Leblanc, 75015 Paris, France; 8 Université Paris Est, Department of Anesthesiology and Critical Care, APHP, Hôpital Henri Mondor, 51 avenue du Marechal de Lattre de Tassigny, 94010 Créteil, France; 9 Department of Anesthesiology and Critical Care, Percy Military Teaching Hospital, 101 avenue Henri-Barbusse, 92140 Clamart, France; 10 AP-HP, Bicêtre Hôpitaux Universitaires Paris Sud, Public Health and Epidemiology Department, AP-HP, Bicêtre Hôpitaux Universitaires Paris Sud, 78 rue du Général Leclerc, F-94275 Le Kremlin Bicêtre, France

**Keywords:** Severe trauma, Severe haemorrhage, Protocol, Organization, Anticipation

## Abstract

**Background:**

Haemorrhagic shock is the leading cause of early preventable death in severe trauma. Delayed treatment is a recognized prognostic factor that can be prevented by efficient organization of care. This study aimed to develop and validate Red Flag, a binary alert identifying blunt trauma patients with high risk of severe haemorrhage (SH), to be used by the pre-hospital trauma team in order to trigger an adequate intra-hospital standardized haemorrhage control response: massive transfusion protocol and/or immediate haemostatic procedures.

**Methods:**

A multicentre retrospective study of prospectively collected data from a trauma registry (Traumabase®) was performed. SH was defined as: packed red blood cell (RBC) transfusion in the trauma room, or transfusion ≥ 4 RBC in the first 6 h, or lactate ≥ 5 mmol/L, or immediate haemostatic surgery, or interventional radiology and/or death of haemorrhagic shock. Pre-hospital characteristics were selected using a multiple logistic regression model in a derivation cohort to develop a Red Flag binary alert whose performances were confirmed in a validation cohort.

**Results:**

Among the 3675 patients of the derivation cohort, 672 (18%) had SH. The final prediction model included five pre-hospital variables: Shock Index ≥ 1, mean arterial blood pressure ≤ 70 mmHg, point of care haemoglobin ≤ 13 g/dl, unstable pelvis and pre-hospital intubation. The Red Flag alert was triggered by the presence of any combination of at least two criteria. Its predictive performances were sensitivity 75% (72–79%), specificity 79% (77–80%) and area under the receiver operating characteristic curve 0.83 (0.81–0.84) in the derivation cohort, and were not significantly different in the independent validation cohort of 2999 patients.

**Conclusion:**

The Red Flag alert developed and validated in this study has high performance to accurately predict or exclude SH.

**Electronic supplementary material:**

The online version of this article (10.1186/s13054-018-2026-9) contains supplementary material, which is available to authorized users.

## Background

Haemorrhage remains the leading cause of early preventable death in severe trauma [1, 2]. A multidisciplinary analysis showed that approximately 2.5% of the deaths in a trauma centre are preventable or potentially preventable. Among the main causes were haemorrhage (39%) and multiple organ failure (28%), often a consequence of haemorrhagic shock. The main reasons for preventable death due to haemorrhage were delayed recognition and management [3]. Organizational optimization is essential to control bleeding as quickly as possible and to reduce patient mortality [4–6]. It is therefore crucial to identify during the pre-hospital phase those patients at high risk of severe haemorrhage (SH) to quickly activate a specific intra-hospital standardized haemorrhage control response, connecting the multispecialty trauma team, blood bank, transfusion protocols, interventional radiology and surgery [7].

Thus, to address efficiently the challenge of SH and shape the response, the design of a haemorrhage specific alert is necessary. Standard triage algorithms are designed to guide severe trauma patients to appropriate trauma centres [8, 9] and trigger trauma team activation [10]. The pre-hospital MGAP score (mechanism, Glasgow coma scale, age and arterial pressure) [11] was developed to predict mortality but showed a proper ability to predict SH (area under the curve 0.7, 95% CI 0.66–0.73) [12]. Established haemorrhage scores predict the need for massive transfusion [13–15]. The TASH score is probably one of the most widely cited scores to predict massive transfusion [16]. However, massive transfusion only applies to a minority of patients, whereas a timely integrative haemostatic strategy could decrease overall transfusion requirements. The aforementioned massive transfusion scores are only validated with intra-hospital data, which renders their application questionable during the pre-hospital phase. A “Code Red” policy has been implemented in trauma centres across the UK [17, 18] with the pre-arrival organization seen as an integral part of the severe haemorrhage pathway [19]. This activation code consists of three criteria (suspicion or evidence of active haemorrhage, systolic arterial blood pressure < 90 mmHg, failure to respond to a fluid bolus) but its predictive accuracy has not yet been evaluated. On a pragmatic standpoint, this type of alert is of utmost importance for trauma centres since emergency care may compete with elective care because of common facilities and workforces.

The aim of our study was to develop and validate an easy-to-use pre-hospital prediction tool for SH in blunt trauma patients derived from a prediction model. This tool is meant to be used as a binary Red Flag alert to activate a specific intra-hospital severe haemorrhage response. The Transparent Reporting of a multivariable prediction model for Individual Prognosis Or Diagnosis (TRIPOD) statement was followed to report its results [20].

## Methods

### Trauma centres and registry

This multicentre observational study used the data collected prospectively from a trauma registry (Traumabase®, traumabase.eu) shared between the six trauma centres of the Paris area in France. These six centres progressively joined the registry between January 2011 and June 2015. Since then, data collection is exhaustive and covers the whole administrative area around Paris, Ile-de-France. The Traumabase® obtained approval from the Advisory Committee for Information Processing in Health Research (CCTIRS, 11.305bis) and from the National Commission on Informatics and Liberties (CNIL, 911461) and meets the requirements of the local and national ethics committee (Comité de Protection des Personnes, Paris VI). The structure of the database integrates algorithms for consistency and coherence. Data monitoring is performed by a central administrator. Sociodemographic, clinical, biological and therapeutic data (from the pre-hospital phase to discharge from the intensive care unit) are systematically collected for all trauma patients. A description of the Emergency Medical System (EMS) and the Trauma System and the characteristics of the Ile-de-France can be found in Hamada et al. [21]. In France, a 24/7 available dispatching physician located in a centralized call centre decides which emergency vector, either a paramedic-staffed ambulance or a physician-staffed mobile intensive care unit, is to be deployed on the basis of the trauma bystanders’ call. Patients brought to dedicated trauma rooms in trauma centres are suspected to be major trauma by the pre-hospital team and are necessarily transported by a physician-staffed ambulance. All patients transported to the trauma rooms of the participating centres were included in the registry (Traumabase®).

Our methodology used a three-step approach: developing a model, deriving a score and transforming the score into a binary alert by choosing the cut-off value.

### Patient selection

**Fig. 1:**
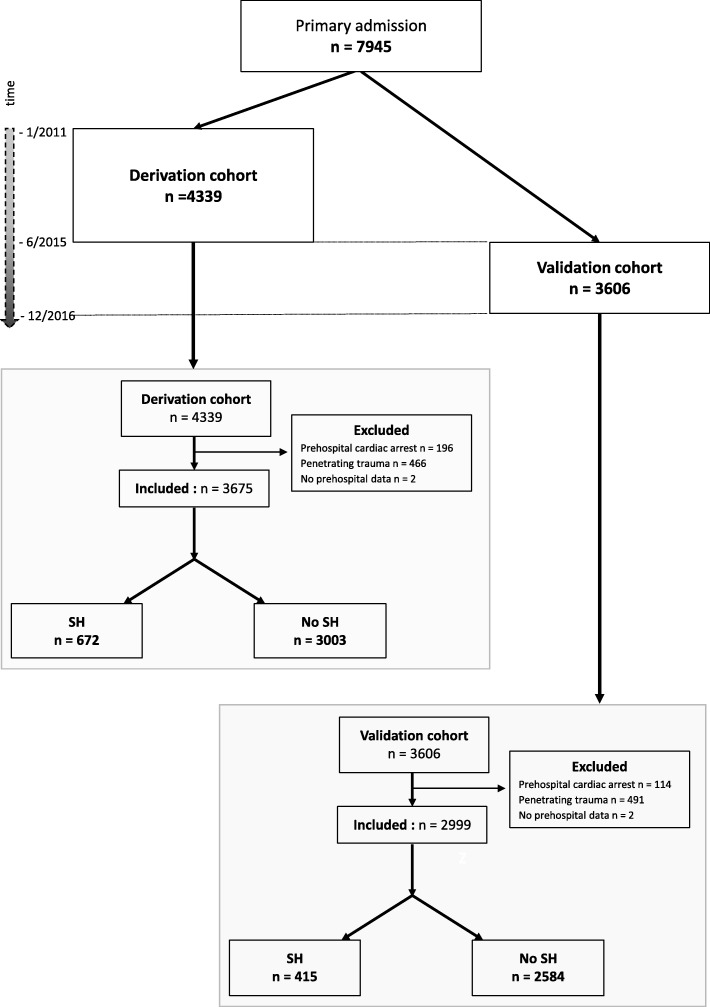
Flowchart of the study. SH severe haemorrhage

Every trauma patient registered in the Traumabase® since January 2011 was included in the study. Patients were excluded if they were admitted after secondary transfer, after penetrating trauma or after pre-hospital traumatic cardiac arrest, or if no pre-hospital data were available. Two sub-samples were constituted: the derivation cohort which included all patients admitted from January 2011 to May 2015, and the validation cohort which included all patients admitted from June 2015 to November 2016 (Fig. 1).

### Definition of severe haemorrhage

Patients were retrospectively considered to present SH on admission to the trauma centre if any of the following criteria was present: need for any packed red blood cell (RBC) transfusion upon arrival in the resuscitation room, transfusion of 4 packed RBCs or more within the first 6 h [22, 23], blood lactate concentration ≥ 5 mmol/L upon arrival [24], need for immediate haemostatic surgery or interventional radiology before complete injury assessment by whole-body CT scan or death from haemorrhagic shock [25]. These criteria were chosen to reflect the heterogeneity and complexity of SH clinical presentation as there is no consensual definition in the literature. The need for an immediate haemostatic intervention was chosen as a reflection of haemorrhage intensity, and for further anticipation of the need of haemostatic resource mobilization. The resuscitation room transfusion criterion and death secondary to haemorrhagic shock were chosen to select the most severe and actively bleeding patients. The transfusion-related criteria represented the dynamic and evolving character of bleeding and need for transfusion over the first hours. Massive transfusion is usually defined as 10 packed RBCs in the first 24 h [26] but this definition is currently questioned [23, 27] and other criteria (transfusion requirements in the first 6 h, transfusion of at least 3 packed RBCs in 1 h or of 5 packed RBCs in 4 h) have been shown to be better correlate with mortality [22, 27]. The transfusion-related criteria were chosen in this study to be a good balance between these different definitions. Finally, the criterion concerning blood lactate level at admission quantified the magnitude of tissue hypoperfusion related to haemorrhage. Apart from metabolic sources for elevated blood lactate level (e.g. alcohol, fast, ethylene glycol), a level higher than 5 mmol/L during the first 24 h is a risk factor for mortality or multiple organ failure [28–30]. For each patient, the presence or absence of SH at admission was adjudicated by SRH, initially blinded to the pre-hospital variables.

### Potential pre-hospital predictors

Thirteen predictors of SH were selected or computed using exclusively pre-hospital data from management on scene and during transport. These criteria were selected based on their clinical significance and ease of use in the pre-hospital time-critical setting: age, sex, minimal systolic, diastolic and mean arterial blood pressure (SBP, DBP and MBP), maximal heart rate (HR), minimal oxygen saturation (SpO_2_), minimal Glasgow Coma Scale (GCS), clinically unstable pelvis, early on-scene point-of-care haemoglobin concentration [31], tracheal intubation and vasopressor administration. The maximal Shock Index was calculated according to the formula: SI = maximal HR / minimal SBP [32].

### Other measured variables

Demographic data, trauma characteristics and outcome were also recorded. For the Simplified Acute Physiology Score (SAPS), the worst value of each variable over the first 24 h was taken into account. The Abbreviated Injury Scale (AIS) version 2005 and the Injury Severity Score (ISS) were calculated when the whole-body injury assessment was completed. The expected probability of survival was calculated using the Trauma and Injury Severity Score (TRISS) with the most recent coefficients [33, 34]. Due to missing data, the TRISS was computed by giving a respiratory rate of 20/min in all patients [35]. The MGAP score was computed as a comparison basis for SH prediction [11].

### Sample size

We used the entire large cohort (7945 patients) as there is no generally accepted approach to estimate the sample size requirements for derivation and validation studies of risk prediction models. The number of events in our sample far exceeds the required number established using previously published rules (10 events per candidate variable for derivation studies and at least 100 events for validation studies) and therefore is expected to provide robust estimates [20].

### Statistical analysis

Continuous data were described as mean ± standard deviation or median (quartiles 1–3) according to their distribution, and categorical variables as count (percentages). The derivation cohort was used to train the prediction model. Univariate analyses were performed to evaluate crude associations between pre-hospital data and the presence of SH using chi-square and Student’s *t* tests (or the Mann–Whitney test when necessary) depending on the variable type. Each variable with *p* < 0.2 was retained as a candidate variable and Spearman correlation coefficients were computed to evaluate collinearity (*r* > 0.8). Candidate continuous variables were dichotomized using receiver operating characteristic (ROC) curves and Youden’s index [36] to identify the cut-off value, except for SpO_2_ for which the clinically relevant cut-off value of ≤ 90% was chosen. Then, candidate binary variables were entered altogether into a multivariate logistic regression model and selection was performed using a backward stepwise procedure to optimize the Akaike criterion. All variables were tested for pairwise univariate interactions. Multiple imputation via a chained equation was used to handle missing data (R PACkage “mice” V 2.3 [37]). A maximum of 5% missing data was observed for the imputed variables. Model calibration was assessed using the Hosmer and Lemeshow statistic [38]. Model discrimination was assessed using the ROC curve (area under the ROC curve (AUC)) and a bootstrap methodology (1000 samples) [39] was used to quantify any optimism (averaged difference between the apparent AUC of the model developed on each bootstrap sample and its AUC on the original sample) in the final prediction model.

To derive the Red Flag binary alert from this model, a score was computed for each patient of the derivation cohort using various combinations of the number of points attributed to each variable that remained in the final prediction model (one point for each variable or two points for variables with a higher model coefficient than the others). Their predictive accuracy was evaluated using the AUC and the Youden’s index was used to identify the cut-off value for the Red Flag binary alert with the best balance between simplicity of use and predictive performance assessed using sensitivity, specificity, positive and negative predictive values (PPV and NPV) and positive likelihood ratio (+LR). Contingency mosaics were drafted for the values around the determined cut-off value.

Finally, the predictive accuracy of the final prediction model and the predictive performance of the resulting Red Flag binary alert were independently assessed in the validation cohort. Model calibration was graphically checked using a calibration plot representing the agreement between risks of SH predicted by the model and observed proportions in the validation cohort.

Discrimination was explored using the AUC ROC. A bootstrap methodology was used to compute the confidence interval (CI) of the AUCs (R package “pROC” V1.10 [40]). The AUC of Red Flag was compared to the AUC of the MGAP score. All tests that were two-sided at *p* ≤ 0.05 were considered significant. R 3.3.3 software (R Foundation for Statistical Computing, Vienna, Austria) was used for analysis.

## Results

The flowchart of both the derivation (*n* = 4339) and validation (*n* = 3606) cohorts is presented in Fig. 1. Patients presenting with initial cardiac arrest, penetrating trauma or no pre-hospital data available were excluded, leaving 3675 (85%) patients for analysis in the derivation cohort and 2999 (83%) in the validation cohort. The distribution of patient across centres is described and illustrated in Additional file 1.

The main characteristics of both cohorts are presented in Table 1. The observed mortality was lower in both groups than the expected mortality according to the TRISS. The two cohorts significantly differed mainly in terms of haemostatic strategies with less surgery (58 vs 51%), more angio-embolization (3% vs 6%) and a shorter intensive care unit length of stay in the validation cohort.

**Table 1:** Derivation and validation cohort characteristics

	Derivation		Validation		Comparison	
	SH (*n* = 672)	No SH (*n* = 3003)	SH (*n* = 415)	No SH (*n* = 2584)	SH *p*	No SH *p*
Demography and outcome						
Age (years)	42 ± 19	37 ± 16	44 ± 19	38 ± 17	ns	ns
Male (%)	465 (69%)	2340 (78%)	312 (75%)	2017 (78%)	0.040	ns
BMI (kg/m^2^)	25.3 ± 5	24.7 ± 4.3	25.2 ± 4.4	24.8 ± 4.7	ns	ns
SAPS II	45 ± 22	21 ± 15	46 ± 23	21 ± 15	ns	ns
ICU LOS	18 ± 23	8 ± 15	14 ± 19	7 ± 15	0.001	0.001
Hospital mortality	163 (25%)	137 (5%)	93 (23%)	110 (4%)	ns	ns
Predicted mortality by TRISS^a^ (%)	27	6	27	6		
Mechanism of injury (all blunt)						
MVA	133 (20%)	765 (26%)	97 (23%)	617 (24%)		
Motorbike	151 (23%)	905 (30%)	95 (23%)	861 (33%)		
Pedestrian and bicycle	109 (16%)	381 (13%)	47 (11%)	343 (13%)	ns	ns
Fall	243 (36%)	792 (26%)	144 (35%)	621 (24%)		
Miscellaneous	36 (5%)	150 (5%)	32 (8%)	142 (6%)		
Severity of injuries						
ISS	30 (18–38)	12 (5–20)	27 (14–41)	12 (5–21)	ns	ns
Head and neck AIS	2 (0–4)	0 (0–3)	1 (0–3)	0 (0–2)	ns	ns
Thorax AIS	3 (0–3)	0 (0–3)	3 (0–4)	0 (0–3)	ns	ns
Abdomen AIS	2 (0–3)	0 (0–2)	2 (0–3)	0 (0–2)	ns	ns
Extremities pelvis AIS	3 (2–3)	2 (0–2)	3 (1–4)	0 (0–3)	ns	0.001
At admission						
Total pre-hospital time (min)	85 ± 39	80 ± 37	80 ± 37	77 ± 34	0.010	ns
SBP (mmHg)	102 ± 34	129 ± 23	107 ± 35	130 ± 24	ns	ns
DBP (mmHg)	62 ± 23	76 ± 16	65 ± 24	79 ± 17	ns	0.001
Haemoglobin (g/dl)	10.2 ± 2.6	13.4 ± 1.7	10.7 ± 2.7	13.5 ± 1.7	0.010	ns
Lactate (mmol/L)	4.8 ± 3.4	1.9 ± 0.9	4.9 ± 3.4	2 ± 0.9	ns	0.002
Prothrombin time (%)	57 ± 22	83 ± 15	61 ± 23	84 ± 15	0.010	ns
Surgery day 1	562 (84%)	1867 (62%)	305 (74%)	1235 (48%)	0.001	0.001
Angio-embolization day 1	111 (17%)	67 (2%)	88 (21%)	86 (3%)	0.001	0.001
SH characteristics						
Immediate surgery	123 (16%)	–	88 (21%)	–	ns	–
Transfusion in trauma room	425 (63%)	–	245 (59%)	–	ns	–
Lactates > 5 mmol/L	323 (51%)	–	194 (52%)	–	ns	–
≥ 4 RBCs in first 6 h	385 (57%)	–	204 (49%)	–	0.010	–

Results expressed as mean ± standard deviation, *n* (%) or median (1st quartile–3rd quartile)

*SH* severe haemorrhage, *ns* not significant, *BMI* body mass index, *SAPS* Simplified Acute Physiology Index, *ICU LOS* intensive care unit length of stay, *TRISS* Trauma Injury Severity Score, *MVA* motor vehicle accident, *ISS* Injury Severity Score, *AIS* Abbreviated Injury Scale, *SBP* systolic blood pressure, *DBP* diastolic blood pressure *RBC* red blood cell

^a^ TRISS computed by giving a respiratory rate of 20/min in all patients [35]

### Score development

Table 2 presents the results of the univariate analysis performed in the derivation cohort (Additional file 2). All of the pre-hospital variables were significantly associated with SH. Collinear variables were HR, SBP, Shock Index (Shock Index was kept), and SBP, DBP and MBP; the latter was kept as not collinear to the Shock Index. As reported in the lower part of Table 2, when there was no clinically relevant cut-off value, Youden’s index was used to dichotomize continuous variables (resulting thresholds are presented in Additional file 3).

**Table 2:** Univariate analysis of pre-hospital variables in the derivation cohort

	SH (*n* = 672)	No SH (*n* = 3003)	Missing values, *n* (%)	*p*
Male	465 (69%)	2258 (78%)	7 (0%)	< 0.001
Age (years)	42 ± 19	37 ± 16	7 (0%)	< 0.001
SBP min (mmHg)	93 ± 30	118 ± 22	50 (1%)	< 0.001
DBP min (mmHg)	55 ± 18	70 ± 15	65 (2%)	< 0.001
MBP min (mmHg)	68 ± 21	86 ± 16	50 (1%)	< 0.001
HR max (/min)	108 ± 27	93 ± 20	73 (2%)	< 0.001
Shock Index (HR/SBP)	1.3 ± 0.8	0.8 ± 0.4	77 (2%)	< 0.001
Capillary haemoglobin (g/dl)	12.8 ± 2.2	14.2 ± 1.7	183 (5%)	< 0.001
SpO_2_ min (%)	97 (92–100)	98 (96–100)	99 (3%)	< 0.001
Glasgow Coma Scale	14 (7–15)	15 (14–15)	17 (0%)	< 0.001
Pelvic trauma	115 (18%)	106 (4%)	141 (5%)	< 0.001
Vasopressor	216 (33%)	140 (5%)	37 (1%)	< 0.001
Pre-hospital intubation	385 (57%)	692 (23%)	6 (0%)	< 0.001
Binarized variables (Youden’s Index)				
SBP min ≤ 100	421 (64%)	569 (19%)		< 0.001
MBP ≤ 70 mmHg	382 (58%)	448 (15%)		< 0.001
HR max ≥100	418 (64%)	1050 (36%)		< 0.001
Shock Index (HR/SBP) ≥ 1	394 (60%)	419 (14%)		< 0.001
Capillary haemoglobin ≤ 13	382 (59%)	812 (29%)		< 0.001
SpO_2_ min ≤ 90%^a^	142 (22%)	189 (6%)		< 0.001
Glasgow Coma Scale ≤13^a^	321 (48%)	712 (24%)		< 0.001

Results expressed as mean ± standard deviation, *n* (%) or median (1st quartile–3rd quartile)

*SH* severe haemorrhage, *SBP* systolic blood pressure, *DBP* diastolic blood pressure, *MBP* mean blood pressure, *HR* heart rate, *SpO*_*2*_ peripheral oxygen saturation, *min* minimal, *max* maximal

^a^Cut-off value not binarized with receiver operating characteristic curves

Ten variables were thus included in the multivariate model and seven variables were selected for the final prediction model (i.e. all but gender, GCS and vasopressor administration) (Table 3). The model goodness-of-fit was good according to the Hosmer–Lemeshow statistic (*p* = 0.60). The discrimination as evaluated by the AUC was 0.84 (95% CI 0.82–0.86). As internal validation, optimism was evaluated at 0.001 using bootstrap methodology, so the optimism-corrected AUC was 0.84 (95% CI 0.82–0.85).

**Table 3:** Results of multivariate stepwise analysis

Pre-hospital criteria	Coefficient	OR	95% CI	*P*
Shock Index > 1	1.32	3.76	2.96–4.78	< 0.001
Pelvic trauma	1.32	3.76	2.68–5.28	< 0.001
Pre-hospital intubation	0.98	2.67	2.17–3.28	< 0.001
Capillary haemoglobin ≤ 13 g/dl	0.92	2.51	2.05–3.08	< 0.001
MBP ≤ 70 mmHg	0.87	2.38	1.88–3.02	< 0.001
Oxygen saturation minimal ≤ 90%	0.59	1.79	1.35–2.39	< 0.001
Age > 50 years	O.42	1.52	1.21–1.92	< 0.001

Model intercept was −3.33 (*p* < 0.0001)

*OR* odds ratio, *CI* confidence interval, *MBP* mean blood pressure

Table 4 presents the predictive performances of several combinations of points attributed to each variable remaining in the final prediction model. The combination associated with the optimal trade-off between predictive accuracy (AUC 0.83 (0.81–0.84)), performance (sensitivity 75% (72–79%), specificity 79% (77–80%)) and ease of use was the simple sum of the following five criteria: SI ≥ 1, point of care haemoglobin ≤ 13 g/dl, pre-hospital intubation, MBP minimum ≤ 70 mmHg and clinical signs of unstable pelvic fracture at any time during pre-hospital management. The Red Flag binary alert was considered to be activated if this score, ranging from 0 to 5, was superior or equal to 2 points. Its predictive performances are presented in Table 4. The three-variable combination (threshold 1 point, Table 4) offers less performance (Delong test for ROC curves of paired data, *p* <  0.001), but seems to be an interesting option especially in a non-physician-staffed EMS (less oro-tracheal intubation and no available pre-hospital haemoglobin measurement).

**Table 4:**
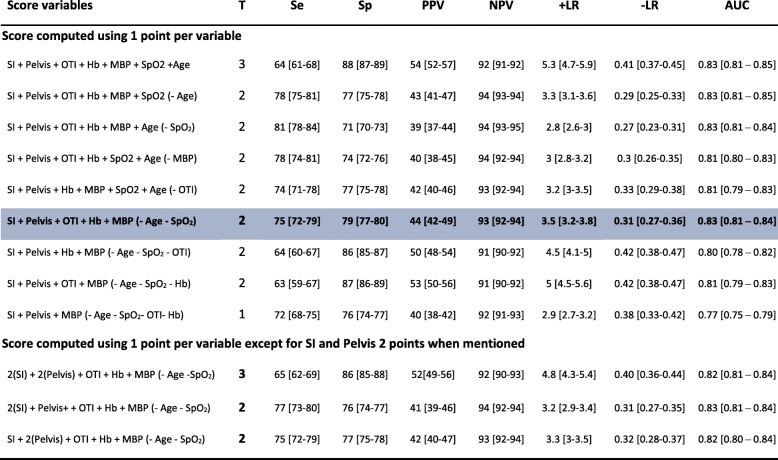
Predictive properties of the various combinations studied to identify the Red Flag binary alert

Shading indicates chosen combination

*T* threshold, *Se* sensitivity, *Sp* specificity, *PPV* positive predictive value, *NPV* negative predictive value, +*LR* positive likelihood ratio, –*LR* negative likelihood ratio, *AUC* area under the receiver operating characteristic curve, *SI* Shock Index, *Pelvis* unstable pelvis, *OTI* oro-tracheal intubation, *Hb* point-of-care haemoglobin, *MBP* mean blood pressure, *SpO*_*2*_ peripheral oxygen saturation

### External validation

**Fig. 2:**
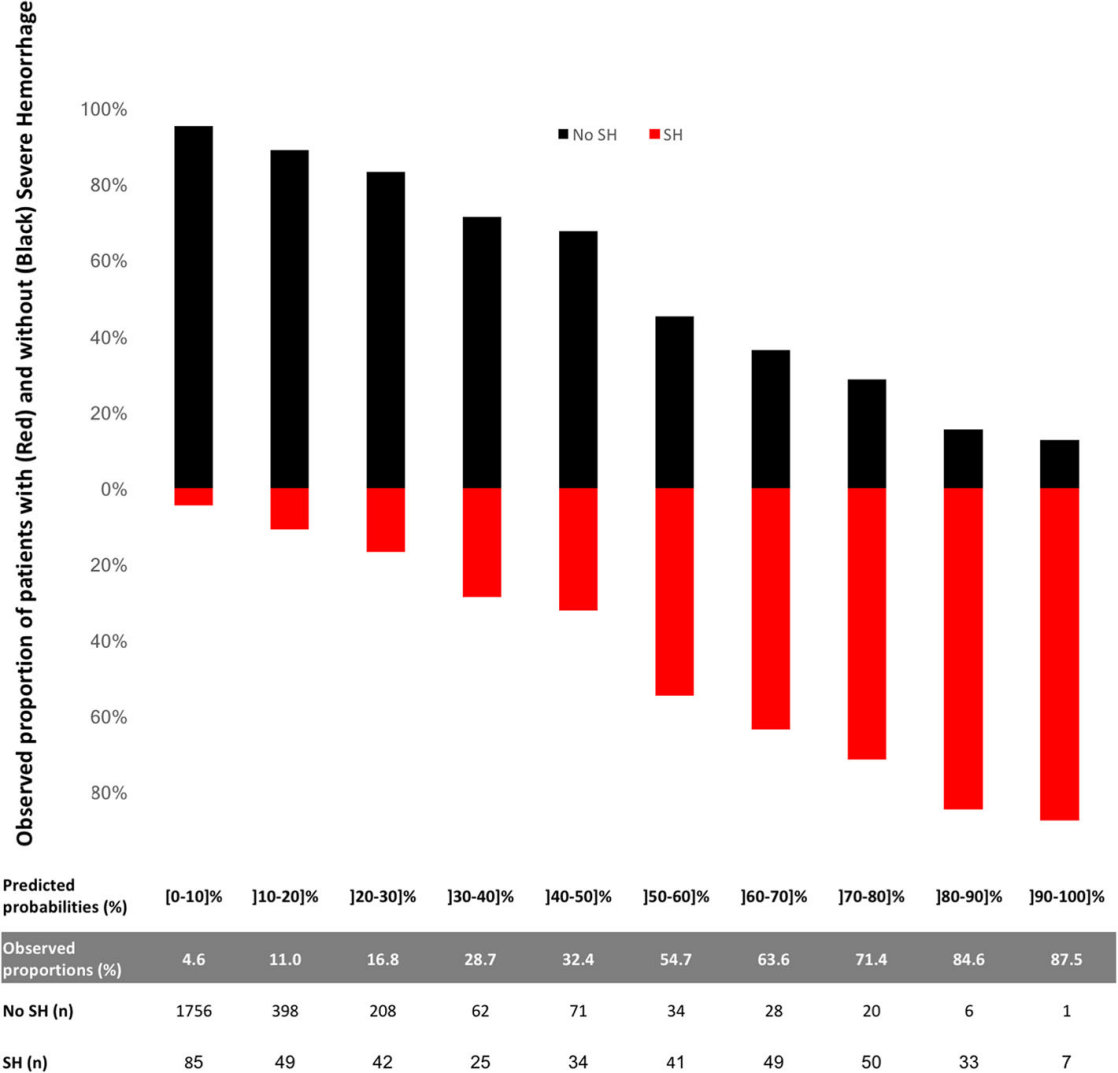
Calibration plot of the model in the validation cohort: agreement between observed and predicted proportion of severe haemorrhage (SH) by the model

**Fig. 3:**
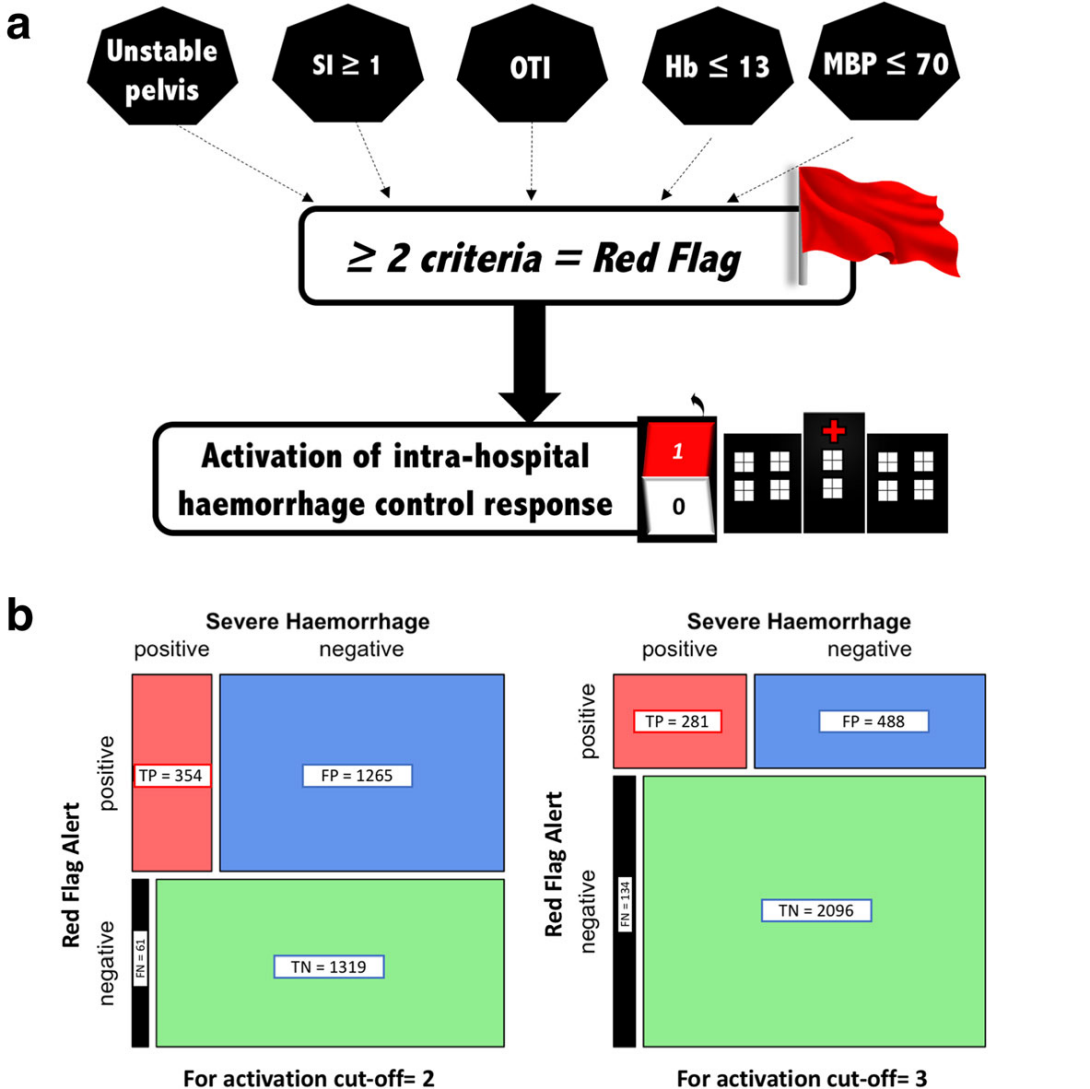
**a** Red Flag alert. **b** Contingency mosaic according to threshold of activation. FN false negative, FP false positive, Hb haemoglobin MBP mean arterial blood pressure, OTI Oro-tracheal intubation, SI Shock Index, TN true negative, TP true positive

Figure 2 shows the calibration plot of the final prediction model in the 2999 patients of the validation cohort. The agreement between predicted probabilities and observed proportions was adequate, except in the group with a predicted risk of SH from 40 to 50% in which the observed proportion of patients with SH was 32%. The AUC of the final prediction model in this population was 0.80 (95% CI 0.78–0.83), similar to the AUC computed in the derivation cohort (*p* = 0.19) and significantly higher than the AUC of the MGAP score equal to 0.72 (95% CI 0.69–0.73) (*p* <  0.001). The predictive performances of the Red Flag binary alert assessed in this population were: sensitivity = 70% (95% CI 66–75), specificity = 80% (95% CI 78–81), PPV = 36% (95% CI 34–38) and NPV = 94% (95% CI 94–95). The contingency mosaics drafted for a threshold of 2 and 3 points are shown in Fig. 3.

## Discussion

In this study, a Red Flag binary alert derived from an efficient combination of pre-hospital criteria was identified with high predictive performances to detect patients at risk of SH. Its high predictive performances were confirmed in internal and external validations. To our knowledge this is the first report of a validated pre-hospital triggered haemorrhage pre-alert. In practice, the presence of any combination of at least two criteria during the pre-hospital care phase among patients with SI (HR/SBP) ≥ 1, unstable pelvic fracture, intubation, point of care haemoglobin ≤ 13 g/dl or MBP ≤ 70 mmHg activates the Red Flag and provides a powerful signal to initiate an adequate intra-hospital standardized haemorrhage control response (massive transfusion protocol and/or immediate haemostatic procedures).

The criteria identified in this study as associated with SH share similarities with some used in previously described haemorrhage control pathways or massive transfusion scores. Unstable pelvic fracture is part of the TASH score, and part of numerous existing scores predicting ongoing haemorrhage [16, 41]. It is a source of internal bleeding that is difficult to control especially in the case of arterial bleeding (20%) but also in the case of venous bleeding, despite pelvic binding. In the TASH score, unstable pelvic fracture accounts for about 20% of the total score (6 points from 28), as in our study (1 from 5). The Shock Index has been demonstrated as a useful sign to diagnose acute hypovolemia and as a good marker of severe haemorrhagic shock [42]. The threshold used in the Red Flag is 1, while the most frequently suggested SI cut-off value to predict massive transfusion is 0.9 in the literature [43]. Also, the threshold of haemoglobin concentration identified in our study was 1 point higher than the threshold used in the TASH score (13 g/dl vs 12 g/dl) [13]. The timing and the technique of measurement used in our study may explain the difference. Indeed, in the present study, haemoglobin concentration was assessed with a point-of-care technique on scene, thus at a very early stage, whereas the TASH score uses the haemoglobin laboratory concentration at hospital admission. Blood pressure is also a key variable in almost all existing predictive scores for severe haemorrhage [16, 41]. Nevertheless, only SBP is used, while the oscillometric sphygmomanometer, used in many EMSs, measures a MBP and extrapolates SBP and DBP via an algorithm [44]. For this reason, MBP was chosen in the Red Flag and the information carried by this variable was found independent of that carried by the SI. Intubation by the pre-hospital team, however, has never been suggested as being associated with severe haemorrhage in previous studies. In our pre-hospital system, physicians are involved in the pre-hospital setting and this may explain this association as it is usually the most severe patients who are intubated during pre-hospital care [45].

The major advantage of this Red Flag alert is its simplicity of use and pragmatism as it is computed with routinely assessed variables and thus directly available criteria for the pre-hospital care team. It allows the rapid identification of patients who require mobilization of important human and material resources to control haemorrhage (advanced immediate resuscitation and/or haemostatic procedures, early and sustained transfusion, etc.). The predictive performances of the previously described “Code Red” have not yet been extensively evaluated [17, 18]. The other existing simple scores are not based on pre-hospital variables [41], or were built to predict outcome such as mortality [11, 46]. The Red Flag could not be compared to the TASH or ABC score. Those latter scores were validated for an intra-hospital setting and include variables such as ultrasound use or blood gas results, variables that are not systematically available in the pre-hospital environment.

The present work is the first to attempt an extensive assessment of the predictive performance of routine pre-hospital data and to include a validation. A code should be easy to remember and the criteria routinely available; both apply to the Red Flag. Indeed, any prediction tool requires evaluation and validation in the very specific setting it will be implemented in. In the case of an inappropriate activation, the complete set of the haemorrhage control infrastructure may be activated and disorganize programmed care for a while. So, any activation code requires a delicate balance between sensitivity and specificity; that is, between the risk of not activating the haemorrhage control pathway when it is needed (false negative, potentially detrimental to the patient) and over-activation (increase in false positive). On the one hand, it is crucial not to miss any haemorrhagic patients and get activated for their arrival, but, on the other, over-activation can generate waste of precious resources and induces team fatigue leading to further non-compliance. Unjustified activation may even reduce the chance of other patients to benefit from the resources inadequately put on standby. An appropriate number of activations, however, maintains team readiness and training. The clinical consequences of this alert will have to be assessed (times, process, outcomes, etc.).

Our study obviously has some limitations. The first is its retrospective design, as it usually precludes the ad hoc choice of the data studied. It might have been interesting to investigate the contribution of other criteria which were not collected in our study: pre-hospital ultrasonography, described as a bleeding characterization criterion in the ABC score [15], or pre-hospital blood lactate dosage described as a predictor of trauma severity [47]. However, this is actually a strength of our study, as it allowed the analysis of pre-hospital data that are routinely collected in practice by the EMS which reinforces the interest in Red Flag as a pragmatic, easy-to-use tool. Moreover, beside this retrospective analysis, data collection was prospective as this study used data from the Traumabase®, and this has limited data loss and biases inherent to retrospective data collection [48]. Furthermore, this study is the first to evaluate the question in a physician-staffed EMS, whereas existing work has been generated in a paramedic-staffed EMS. Transposition of experiences and data from one system to another can be difficult. The external validity of the study could only be assessed by testing and validating it outside the original centres. The characteristics of these centres, however, are quite different with regard to equipment, internal organization and case mix as there is a lot of contrast in demographic characteristics of the different area covered by each centre within the region. So, results from this study are thus likely to be transposable to other centres. Furthermore, demographic and clinical characteristics of our cohort, as well as mortality, were similar to those in the trauma literature [14, 49]. It is noteworthy that this alert does not apply to penetrating trauma and has not been validated for children. Finally, the impact of this Red Flag alert on patient care has not been evaluated and requires a separate prospective study.

## Conclusion

We have constructed and validated a simple Red Flag alert for identifying severe blunt trauma patients during the pre-hospital care phase and activating a specific immediate intra-hospital haemorrhage control response prior to arrival. The impact of its use on severe trauma patient care and on resource utilization remains to be determined.

## Additional files

Additional file 1: Distribution of origin of patients for derivation and validation cohorts. (TIFF 14113 kb)

Additional file 2: Binarization of continuous variables according to Youden’s Index. SpO_2_ binarized according to literature (cut-off value 90%). AUC area under the ROC curve. (DOCX 16 kb)

Additional file 3: Univariate analysis of pre-hospital variables in derivation cohort. Results expressed as mean ± standard deviation or *median [1st quartile–3rd quartile]. SBP systolic blood pressure, DBP diastolic blood pressure, MBP mean blood pressure, HR heart rate, SpO_2_ peripheral oxygen saturation, Min minimal, Max maximal. #Cut-off value not binarized with ROC curves. (DOCX 19 kb)

## Abbreviations

+LR: Positive likelihood ratio
AIS: Abbreviated Injury Scale
AUC: Area under the ROC curve
BMI: Body mass index
DBP: Diastolic arterial blood pressure
EMS: Emergency Medical Service
GCS: Glasgow Coma Scale
Hb: Haemoglobin
ICU LOS: Intensive care unit length of stay
ISS: Injury Severity Score
MBP: Mean arterial blood pressure
MGAP: Mechanism, Glasgow, age and arterial pressure
MVA: Motor vehicle accident
NPV: Negative predictive value
OTI: Oro-tracheal intubation
POC: Point of care
PPV: Positive predictive value
ROC: Receiver operating characteristic
SAPS: Simplified Acute Physiology Index
SBP: Systolic arterial blood pressure
Se: Sensitivity
SH: Severe haemorrhage
SI: Shock Index
Sp: Specificity
SpO_2_: Peripheral oxygen saturation
TRISS: Trauma Injury Severity Score
