# Supplementary figures and images for: Development and validation of a pre-hospital “Red Flag” alert for activation of intra-hospital haemorrhage control response in blunt trauma
Source: Crit Care. 2018 May 5;22:113. doi: 10.1186/s13054-018-2026-9 (PMC5935988; doi:10.1186/s13054-018-2026-9)

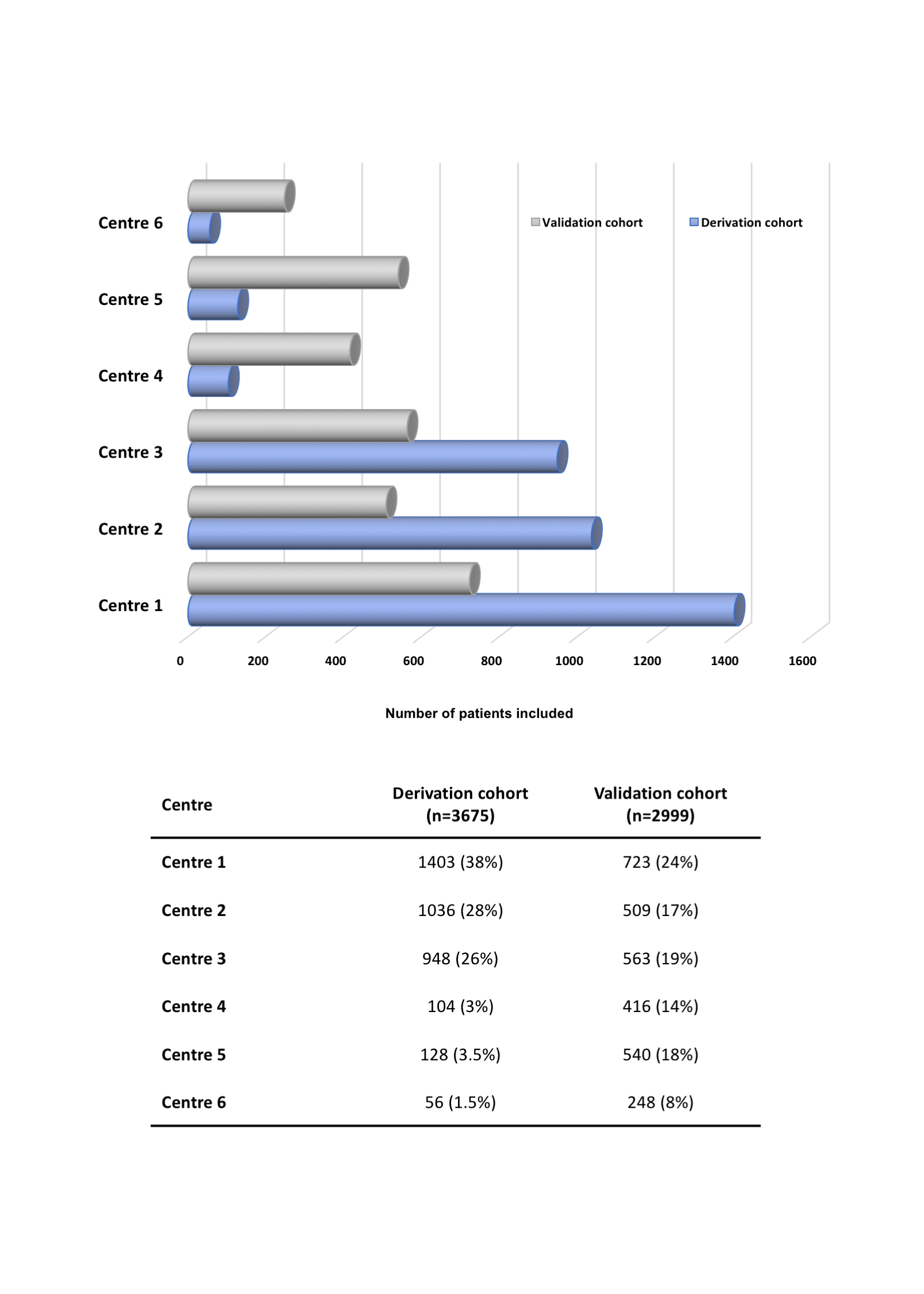

Supplement: Supplementary file 1 — Distribution of origin of patients for derivation and validation cohorts. (TIFF 14113 kb) [file 13054_2018_2026_MOESM1_ESM.tiff]
