# Supplementary material for: Development and validation of a pre-hospital “Red Flag” alert for activation of intra-hospital haemorrhage control response in blunt trauma
Source: Crit Care. 2018 May 5;22:113. doi: 10.1186/s13054-018-2026-9 (PMC5935988; doi:10.1186/s13054-018-2026-9)
Supplement: Supplementary file 2 — Binarization of continuous variables according to Youden’s Index. SpO2 binarized according to literature (cut-off value 90%). AUC area under the ROC curve. (DOCX 16 kb) [file 13054_2018_2026_MOESM2_ESM.docx]

**Additional file 3**. ***Table S2 bis***. Univariate analysis of the prehospital variables in the derivation cohort

|  | **HS group**  **(n=415)** | **No SH group**  **(n=2584)** | **Missing values**  **(%)** | **p** |
| --- | --- | --- | --- | --- |
| **Male, n (%)** | 312 (75%) | 2017 (78%) | 5 (0%) | 0.19 |
| **Age (year)** | 44 ± 19 | 38 ± 17 | 1 (0%) | < 0.001 |
| **SBP min (mmHg)** | 94 ± 26 | 118 ± 21 | 83 (3%) | < 0.001 |
| **DBP min (mmHg)** | 56 ± 18 | 71 ± 15 | 88 (3%) | < 0.001 |
| **MBP min (mmHg)** | 69 ± 20 | 86 ± 16 | 83 (3%) | < 0.001 |
| **HR max (/min)** | 111 ± 26 | 93 ± 20 | 91 (3%) | < 0.001 |
| **Shock Index (HR/SAP)** | 1.3 ± 0.6 | 0.8 ± 0.3 | 101 (3%) | < 0.001 |
| **Capillary Haemoglobin (g/dl)** | 10.9 ± 2.8 | 14.1 ± 1.8 | 228 (8%) | < 0.001 |
| **Oxygen saturation min* (%)** | 97 [92 - 99] | 98 [95 - 100] | 74 (3%) | < 0.001 |
| **Glasgow Coma Scale*** | 14 [8 - 15] | 15 [14 - 15] | 14 (1%) | < 0.001 |
| **Pelvic trauma, n (%)** | 81 (20%) | 81 (3%) | 11 (0%) | < 0.001 |
| **Vasopressor, n (%)** | 134 (35%) | 112 (4%) | 117 (4%) | < 0.001 |
| **Prehospital intubation, n (%)** | 212 (51%) | 489 (19%) | 10 (0%) | < 0.001 |
| ***Binary variables (Youden)*** |  |  |  |  |
| **- SBP min ≤ 100, n (%)** | 257 (64%) | 491 (20%) |  | < 0.001 |
| **- MBP ≤ 70 mmHg, n (%)** | 225 (56%) | 374 (15%) |  | < 0.001 |
| **- HR max ≥ 100, n (%)** | 264 (66%) | 898 (36%) |  | < 0.001 |
| **- Shock Index (HR/SAP) ≥1 (%)** | 245 (62%) | 376 (15%) |  | < 0.001 |
| **- Capillary Haemoglobin ≤ 13, n (%)** | 194 (50%) | 629 (26%) |  | < 0.001 |
| **- SpO_2_ min ≤ 90%, n (%) *^#^*** | 86 (22%) | 186 (7%) |  | < 0.001 |
| **- Glasgow Coma Scale ≤ 13, n (%) *^#^*** | 160 (39%) | 510 (20%) |  | < 0.001 |

Results expressed as mean ± standard deviation or *Median [1^st^ quartile-3^rd^ quartile]. Systolic Blood Pressure, DBP: Diastolic Blood Pressure, MBP: Mean Blood Pressure, HR: heart rate, SpO_2_: peripheral oxygen saturation, Min: Minimal, Max: Maximal.

#: cut-off not binarized with ROC curves
