# Supplementary material for: Development and validation of a pre-hospital “Red Flag” alert for activation of intra-hospital haemorrhage control response in blunt trauma
Source: Crit Care. 2018 May 5;22:113. doi: 10.1186/s13054-018-2026-9 (PMC5935988; doi:10.1186/s13054-018-2026-9)
Supplement: Supplementary file 3 — Univariate analysis of pre-hospital variables in derivation cohort. Results expressed as mean ± standard deviation or *median [1st quartile–3rd quartile]. SBP systolic blood pressure, DBP diastolic blood pressure, MBP mean blood pressure, HR heart rate, SpO2 peripheral oxygen saturation, Min minimal, Max maximal. #Cut-off value not binarized with ROC curves. (DOCX 19 kb) [file 13054_2018_2026_MOESM3_ESM.docx]

**Additional file 2**. Binarisation of continuous variables according to Youden’s Index

| **Variable** | **Best threshold** | **Sensitivity** | **Specificity** | **AUC** |
| --- | --- | --- | --- | --- |
| **Age (years)** | 50 | 34% | 78% | 0.58 |
| **Systolic Blood Pressure (mmHg)** | 100 | 70% | 77% | 0.76 |
| **Mean Blood Pressure (mmHg)** | 70 | 57% | 85% | 0.76 |
| **Heart rate** | 100 | 65% | 54% | 0.68 |
| **Shock index** | 1 | 70% | 77% | 0.78 |
| **Capillary Haemoglobin (g/ dL)** | 13 | 56% | 72% | 0.69 |
| **Glasgow Coma Scale** | 13 | 45% | 78% | 0.64 |

AUC: Area Under the ROC Curve, SpO_2_ was binarised according to literature (cut-off 90%)
